# Distinct cardioprotective mechanisms of immediate, early and delayed ischaemic postconditioning

**DOI:** 10.1007/s00395-014-0452-7

**Published:** 2014-12-02

**Authors:** Veronika Barsukevich, Marina Basalay, Jenifer Sanchez, Alexander Mrochek, John Whittle, Gareth L. Ackland, Alexander V. Gourine, Andrey Gourine

**Affiliations:** 1 Research Centre Cardiology, Minsk, Belarus; 2 Neuroscience, Physiology and Pharmacology, University College London, London, UK; 3 Department of Medicine, University College London, London, UK; 4 Department of Cardiology, Karolinska University Hospital, Stockholm, Sweden

**Keywords:** Ischaemia and reperfusion injury, MitoK_ATP_ channels, Myocardial infarction, Postconditioning, Preconditioning, RISK and SAFE pathways

## Abstract

Cardioprotection against ischaemia/reperfusion injury in mice can be achieved by delayed ischaemic postconditioning (IPost) applied as late as 30 min after the onset of reperfusion. We determined the efficacy of delayed IPost in a rat model of myocardial infarction (MI) and investigated potential underlying mechanisms of this phenomenon. Rats were subjected to 20, 30 or 45 min of coronary artery occlusion followed by 120 min of reperfusion (I/R). Immediate and early IPost included six cycles of I/R (10/10 s) applied 10 s or 10 min after reperfusion onset. In the second series of experiments, the rats were subjected to 30 min of coronary occlusion followed by IPost applied 10 s, 10, 30, 45 or 60 min after the onset of reperfusion. Immediate and early IPost (applied 10 s or 10 min of reperfusion) established cardioprotection only when applied after a period of myocardial ischaemia lasting 30 min. Delayed IPost applied after 30 or 45 min of reperfusion reduced infarct sizes by 36 and 41 %, respectively (both *P* < 0.01). IPost applied 60 min after reperfusion onset was ineffective. Inhibition of RISK pathway (administration of ERK1/2 inhibitor PD-98059 or PI3K inhibitor LY-294002) abolished cardioprotection established by immediate IPost but had no effect on cardioprotection conferred by early IPost. Blockade of SAFE pathway using JAK/STAT inhibitor AG490 had no effect on the immediate or early IPost cardioprotection. Blockade of mitochondrial K_ATP_ (mitoK_ATP_) channels (with 5-Hydroxydecanoate) abolished cardioprotection achieved by immediate and early IPost, but had no effect on cardioprotection when IPost was applied 30 or 45 min into the reperfusion period. Immediate IPost increased phosphorylation of PI3K-AKT and ERK1/2. Early or delayed IPost had no effect on phosphorylation of PI3K-AKT, ERK1/2 or STAT3. These data show that in the rat model, delayed IPost confers significant cardioprotection even if applied 45 min after onset of reperfusion. Cardioprotection induced by immediate and early postconditioning involves recruitment of RISK pathway and/or mitoK_ATP_ channels, while delayed postconditioning appears to rely on a different mechanism.

## Introduction

Infarct size is the major determinant of prognosis in patients with an acute myocardial infarction (MI). The landmark experimental study by Reimer et al. [34] described the wavefront phenomenon of myocardial cell death by showing that infarct size is dependent upon the duration of myocardial ischaemia. Restitution of blood supply to an ischaemic area is, therefore, crucial for tissue survival but it also results in a cascade of harmful events known as lethal reperfusion injury [29]. The latter is defined as the death of cardiomyocytes that are still viable at the end of the ischaemic period. Clinical therapies aimed to reduce the extent of myocardial ischaemia/reperfusion injury are still limited to thrombolysis or immediate percutaneous coronary intervention. In experimental studies, Zhao et al. [51] used a dog model of MI and demonstrated that the infarct size could be significantly reduced when three brief cycles of ischaemia/reperfusion (30/30 s) are applied after the onset of reperfusion which followed a 60 min period of ischaemia. This immediate ischaemic postconditioning (IPost) was also found to be effective in conferring cardioprotection in isolated hearts and different animal models [40]. However, there are also discordant reports showing that IPost fails to establish cardioprotection [6, 26, 38, 40]. Human studies assessing the efficacy of immediate IPost demonstrated significant reduction [25, 27, 46, 47], modest [41] or no effect [7, 24, 45] of IPost on infarct size.

The time window for protective intervention(s) during reperfusion has important implications in terms of understanding the mechanisms underlying myocardial ischaemia/reperfusion injury and clinical usefulness of IPost. In support of the prevailing concept, stating that any cardioprotective strategy should be applied immediately after the onset of reperfusion [32], Kin et al. [22] demonstrated in a rat model of MI that IPost is not effective when applied 1 min into the reperfusion period. However, recent data obtained in mice suggest that cardioprotection can still be established by delayed IPost applied as late as 30 min after reperfusion onset [35]. While rejecting the idea of an instantaneous reperfusion injury, Roubille et al. [35] proposed the theory of a dynamic wavefront of reperfusion-induced cell death which develops over a certain period of time during reperfusion period.

Endogenous mechanisms of cardioprotection recruited early in reperfusion by immediate IPost involve activation of Reperfusion Injury Salvage Kinase (RISK) and/or Survival Activating Factor Enhancement (SAFE) signalling pathways leading to inhibition of the mitochondrial permeability transition pore (mPTP) [13, 18] and activation of mitochondrial ATP-dependent potassium (mitoK_ATP_) channels [18]. RISK pathway includes pro-survival kinase cascades among which mitogen-activated protein kinase p44/p42 (ERK1/2) and phosphatidylinositol 3-kinase AKT (PI3K/AKT) play important roles [14]. SAFE pathway includes tumour necrosis factor-α receptors and janus-activated kinase (JAK) signal transducer and activator of transcription (STAT) [23].

In this study, we determined the efficacy of delayed IPost in a rat model of MI (with various ischaemia durations) and investigated potential underlying mechanisms of this phenomenon by pharmacological blockade of mitoK_ATP_ channels, RISK and SAFE pathways, as well as Western blot analysis of RISK and SAFE activation.

## Methods

All the experiments were performed in accordance with the European Commission Directive 86/609/EEC (European Convention for the Protection of Vertebrate Animals used for Experimental and Other Scientific Purposes) and the UK Home Office (Scientific Procedures) Act (1986) with project approval from the respective Institutional Animal Care and Use Committees.

### Animal preparation

Adult male Wistar rats (280–320 g) were anaesthetized with pentobarbital sodium (induction 60 mg kg^−1^ i.p.; maintenance 10–15 mg kg^−1^ h^−1^ i.v.). Adequate anaesthesia was ensured by maintaining stable levels of arterial blood pressure and heart rate and confirmed by the absence of a withdrawal response to a paw pinch. The right carotid artery and left jugular vein were cannulated for measurement of the arterial blood pressure and administration of anaesthetic or test compounds, respectively. The trachea was cannulated, and the animal was ventilated with room air using a positive pressure ventilator with a tidal volume of ~8–10 ml kg^−1^ and a ventilator frequency of ~60 strokes min^−1^. A standard lead II ECG was recorded and the body temperature was maintained at 37.0 ± 0.2 °C.

### Model of myocardial infarction

The heart was exposed via a left thoracotomy and a 5–0 monofilament polypropylene suture was passed around the left anterior descending coronary artery (LAD) to induce occlusion(s). Myocardial ischaemia lasting 20, 30 or 45 min was induced by LAD ligation and was followed by 120 min of reperfusion.

### Measurements of infarct size

At the end of the reperfusion period, the LAD was re-occluded, and Evans Blue dye solution was injected into the jugular vein for the assessment of the area at risk. The animal was then given an anaesthetic overdose, the heart was excised, and the left ventricle (LV) was isolated, frozen and sectioned into 5–6 transverse slices from apex to the base. The area at risk was demarcated by the absence of Evans Blue staining. LV slices were then incubated with 1 % 2,3,5-triphenyltetrazolium chloride (TTC) in Tris buffer (pH 7.4) for 15 min at 37 °C and fixed (4 % formalin). Viable myocardium is stained red by TTC, whereas necrotic myocardium appears pale yellow. The area at risk and the necrotic area were determined by computerised planimetry, normalised to the weight of each slice, with the degree of necrosis (i.e. infarct size) expressed as the percentage of area at risk.

### Experimental protocols

- Protocol 1. To determine the efficacy of immediate, early and delayed IPost in conferring cardioprotection against myocardial ischaemia/reperfusion injury and its dependence upon the duration of the ischaemic period

**Fig. 1:**
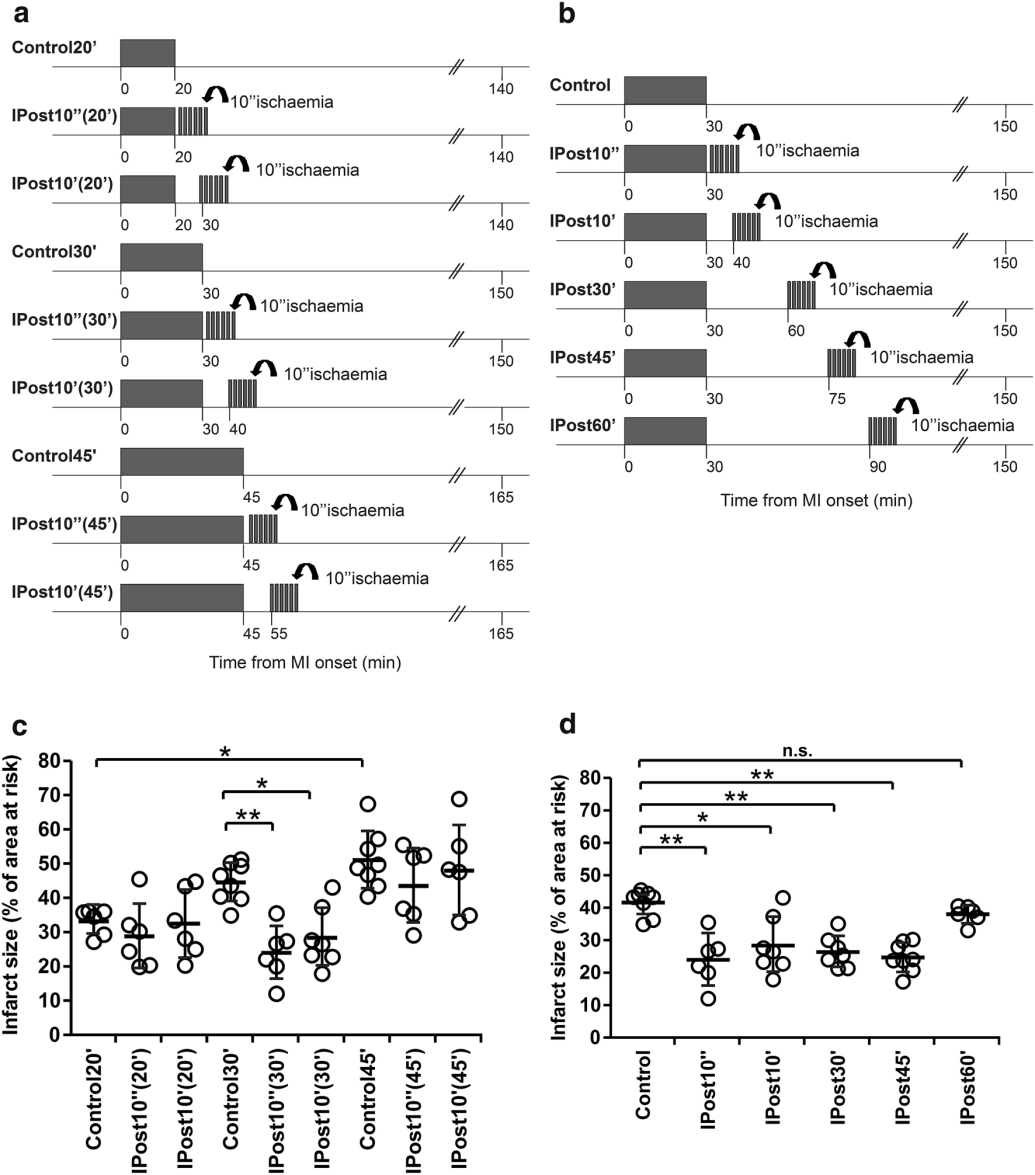
**a** Illustration of the experimental protocols. Myocardial infarction (MI) was induced in anaesthetized rats by left descending coronary artery (LAD) occlusion followed by 120 min of reperfusion (I/R). Ischaemic postconditioning (IPost) included six cycles of I/R (10 s/10 s) starting 10 s (IPost10″) or 10 min (IPost10′) after the onset of reperfusion which followed periods of LAD occlusion lasting either 20 min [IPost10″(20′), IPost10′(20′)], 30 min [IPost10″(30′), IPost10′(30′)] or 45 min [IPost10″(45′), IPost10′(45′)]. **b** Illustration of the experimental protocols. MI was induced by 30 min of LAD occlusion followed by 120 min of reperfusion. IPost was applied 10 s (IPost10″), 10 min (IPost10′), 30 min (IPost30′), 45 min (IPost45′) or 60 min (IPost60′) after the onset of reperfusion. **c** IPost effectively reduces myocardial ischaemia/reperfusion injury only when applied after a period of LAD occlusion lasting 30 min. **d** IPost reduces myocardial ischaemia/reperfusion injury when applied 10 s, 10, 30 or 45 min after the onset of reperfusion which followed periods of LAD occlusion lasting 30 min. Infarct sizes are presented as percentages of the areas at risk. Individual data and mean ± SD are shown. **P < 0.01; *P < 0.05; n.s.—not significant

Myocardial ischaemia/reperfusion injury was induced by either 20 min (*n* = 6), 30 min (*n* = 8), or 45 min (*n* = 8) of LAD occlusion followed by 120 min of reperfusion (I/R). Immediate and early IPost included six cycles of I/R (10 s/10 s) starting: 10 s (IPost10″) or 10 min (IPost10′) following periods of ischaemia lasting either 20 min (*n* = 6), 30 min (*n* = 7) or 45 min (*n* = 6), respectively (Fig. 1a). To determine the efficacy of delayed IPost in this model, six cycles of I/R (10 s/10 s) were applied 30 min (IPost30′, *n* = 7), 45 min (IPost45′, *n* = 8) or 60 min (IPost60′, *n* = 6) after the onset of reperfusion which followed period of ischaemia lasting 30 min (Fig. 1b).

- Protocol 2. To investigate the role of RISK and SAFE pathways in cardioprotection established by IPost.

**Fig. 2:**
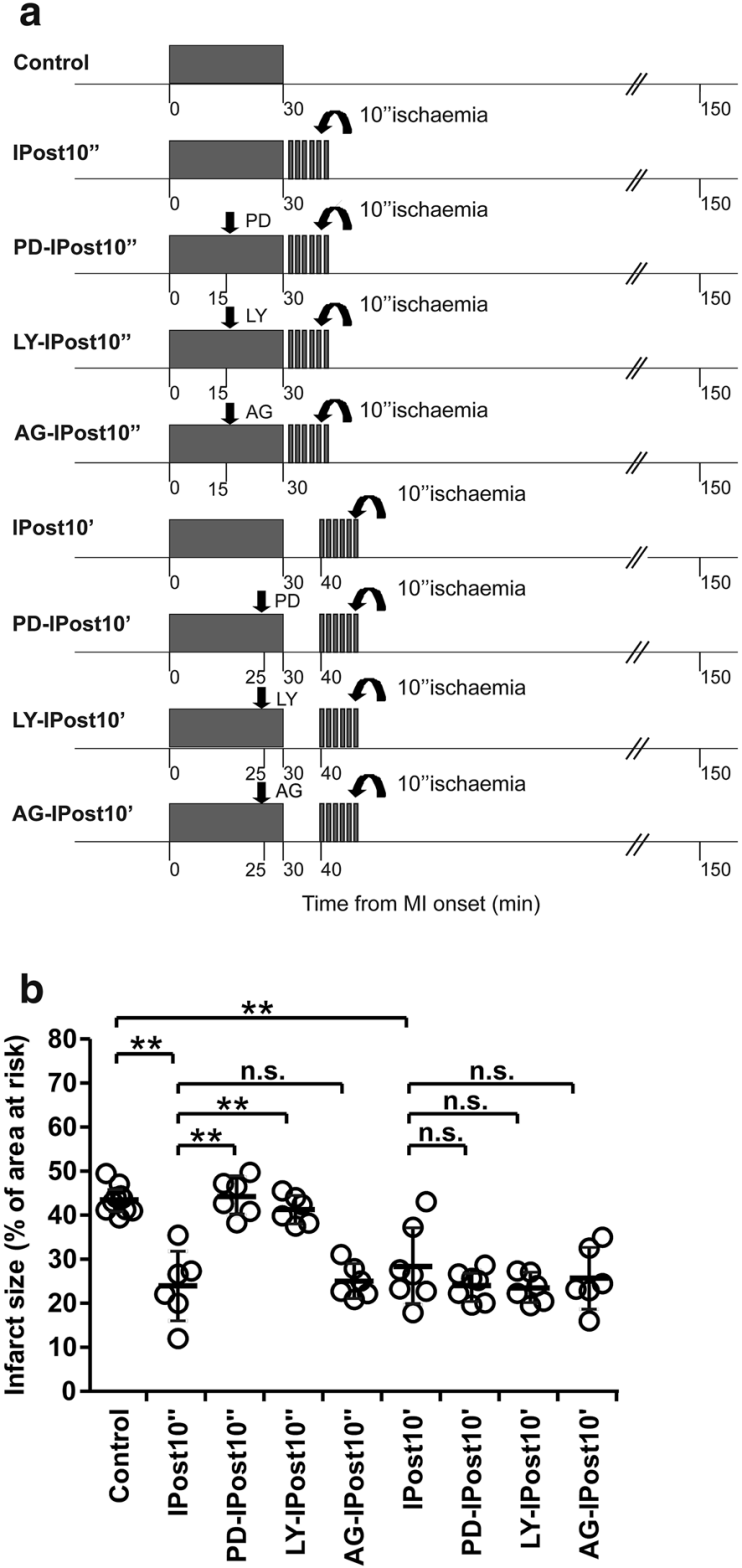
**a** Illustration of the experimental protocols. *Arrow* indicates time of intravenous administration of the inhibitors of RISK [PD-98059 (PD), LY-294002 (LY)] or SAFE pathways [AG490 (AG)]. **b** PD and LY abolished cardioprotection established by immediate IPost (IPost10″), but had no effect on cardioprotection conferred by early IPost (IPost10′). AG had no effect on the immediate or early IPost cardioprotection. Infarct sizes are presented as percentages of the areas at risk. Individual data and mean ± SD are shown. **P < 0.01; *P < 0.05; n.s.—not significant

Myocardial ischaemia/reperfusion injury was induced by 30 min (*n* = 10) of LAD occlusion followed by 120 min of reperfusion. The control group received vehicle (5 % DMSO in saline) injected i.v. 15 min before reperfusion. Immediate and early IPost protocols included six cycles of I/R (10 s/10 s) starting, respectively, 10 s (IPost10″, *n* = 6) or 10 min (IPost10′, *n* = 7) after the onset of reperfusion (Fig. 2a). ERK1/2 inhibitor PD-98059 (0.3 mg kg^−1^, i.v.) was administered 15 or 25 min after the start of the ischaemic period in animals subjected to IPost applied 10 s (PD-IPost10″ group; *n* = 6) or 10 min after reperfusion onset (PD-IPost10′ group; *n* = 7), respectively. PI3K inhibitor LY-294002 (0.3 mg kg^−1^, i.v.) was administered 15 or 25 min after the start of the ischaemic period in animals subjected to IPost applied 10 s (LY-IPost10″ group, *n* = 6) or 10 min after reperfusion onset (LY-IPost10′ group, *n* = 6), respectively. JAK/STAT pathway inhibitor AG490 (3 mg kg^−1^, i.v.) was administered 15 or 25 min after the start of the ischaemic period in animals subjected to IPost applied 10 s (AG-IPost10″ group, *n* = 6) or 10 min after reperfusion onset (AG-IPost10′ group, *n* = 6), respectively. The doses and timings of PD-98059, LY-294002 and AG490 applications were selected on the basis of previously published studies [43, 48].

### Immunoblotting for survival kinases

In an additional set of experiments, rats underwent 30 min of myocardial ischaemia and the hearts were collected at 15 min after sham-IPost or IPost (six cycles of I/R, 10 s/10 s) applied 10 s, 10 min or 45 min after reperfusion onset. Rats were randomly assigned to one of the following six groups: sham-immediate IPost (sham-IPost10″, *n* = 6), immediate IPost (IPost10″, *n* = 6), sham-early IPost (sham-IPost10′, *n* = 6), early IPost (IPost10′, *n* = 6), sham-delayed IPost (sham-IPost45′, *n* = 6) and delayed IPost, (IPost45′, *n* = 6). For the analysis of protein phosphorylation, Western blots were performed on myocardium from the area at risk. The ventricular tissue was excised, frozen in liquid nitrogen and stored at −80 °C before the assays. Total AKT, p42/p44 MAPK (ERK1/2), STAT3 and their respective phospho-proteins were immunodetected from cell lysates using specific primary antibodies (all from Cell Signalling Technology, New England Biolabs, Hitichin, UK). Proteins were resolved on SDS-PAGE gels and transferred to polyvinylidene difluoride membranes (Amersham Biosciences, Piscataway, USA) according to the manufacturer’s instructions. After antibody labelling, detection was performed (ECL detection system, Amersham Biosciences, Piscataway, NJ). Densitometry was used to calculate the ratio of phosphorylated and total protein in IPost and respective sham groups, normalised to the expression of β-actin or CDK4 protein (Santa Cruz, Insight Biotechnology, Wembley, UK or Cell Signalling Technology, New England Biolabs, Hitichin, UK) to control protein loading.

- Protocol 3. To investigate the role of mitoK_ATP_ channels in cardioprotection established by IPost.

Myocardial ischaemia/reperfusion injury was induced by 30 min (*n* = 8) of LAD occlusion followed by 120 min of reperfusion. Immediate, early and delayed IPost protocols included six cycles of I/R (10 s/10 s) starting, respectively, 10 s (IPost10″, *n* = 6), 10 min (IPost10′, *n* = 7), 30 min (IPost30′, *n* = 9) or 45 min (IPost45′, *n* = 8) after reperfusion onset (Fig. 4a). The mitoK_ATP_ channel blocker 5-hydroxydecanoate (5-HD, 10 mg kg^−1^, i.v.) was administered 20 or 29 min after the start of the ischaemia period in animals subjected to IPost applied 10 s (5-HD-IPost10″ group; *n* = 6) or 10 min after reperfusion onset (5-HD-IPost10′ group; *n* = 6), respectively. To determine the role of mitoK_ATP_ channels in cardioprotection established by delayed IPost, 5-HD was given 20 or 35 min after the end of the ischaemia period in animals subjected to IPost applied 30 or 45 min after reperfusion onset, respectively (5-HD-IPost30′, *n* = 6 and 5-HD-IPost45′, *n* = 6; groups). The dose and timings of 5-HD administration were selected on the basis of previously published reports [11].

### Statistical analysis

Data are reported as mean ± SD. Data were compared by ANOVA followed by Tukey–Kramer post hoc test or unpaired *t* test, as appropriate. Values of *P* < 0.05 were considered to be significant.

## Results

No differences in mean arterial blood pressure or heart rate before or during ischaemia and reperfusion were observed between groups of animals recruited into the experimental protocols (data not shown). There were also no differences in the areas at risk between experimental groups (mean values of areas at risk ranged between 39 and 43 %). Figures 1c, d, 2b and 4b illustrate infarct sizes expressed as percentages of the area at risk.

### Efficacy of immediate, early and delayed IPost in conferring cardioprotection against myocardial ischaemia/reperfusion injury and its dependence upon the duration of the ischaemic period.

Infarct sizes in animals subjected to 20, 30 and 45 min of LAD occlusion followed by 120 min of reperfusion were 33 ± 3, 44 ± 8, and 51 ± 8 %, respectively (Fig. 1c). Infarcts in animals subjected to 45 min of ischaemia were significantly larger than in animals subjected to 20 min of ischaemia (*P* < 0.05) (Fig. 1c). IPost reduced infarct sizes by 43 % (*P* < 0.01) and 31 % (*P* < 0.05) when applied either 10 s or 10 min after the end of myocardial ischaemia which had the duration of 30 min (Fig. 1c). IPost failed to reduce the infarct size when applied 10 s or 10 min following periods of myocardial ischaemia lasting either 20 or 45 min (Fig. 1c). Delayed IPost applied either 30 min or 45 min after the onset of reperfusion effectively reduced infarct size by 36 % (*P* < 0.01) and 41 % (*P* < 0.01), respectively (Fig. 1d). IPost applied after 60 min of reperfusion was ineffective (Fig. 1d).

### The role of RISK and SAFE pathways in cardioprotection established by IPost

**Fig. 3:**
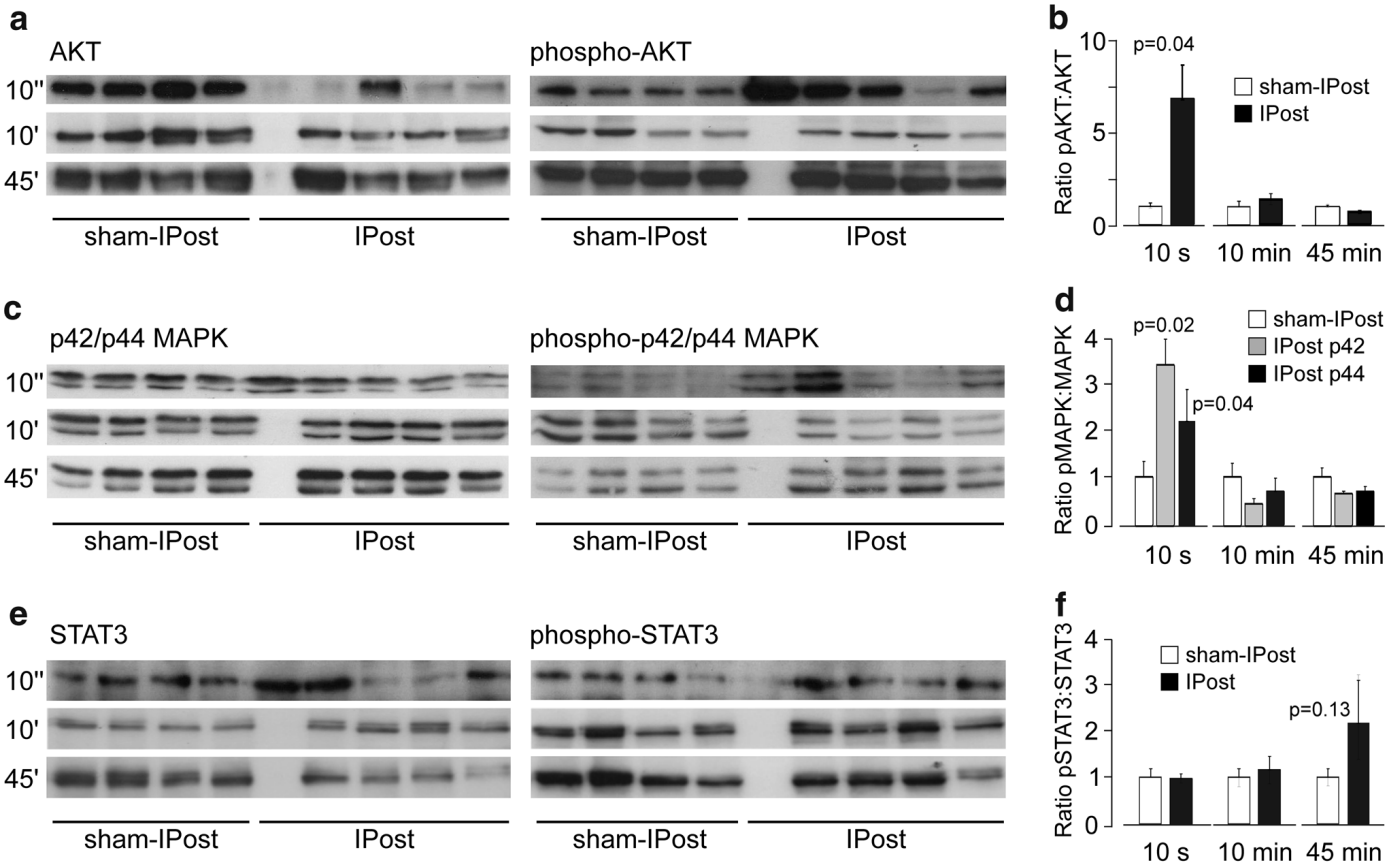
**a** Representative immunoblots showing total AKT and phospho-AKT (Ser473) protein expression in left ventricular lysates following immediate IPost (10 s after reperfusion onset), early IPost (10 min after reperfusion onset), delayed IPost (45 min after reperfusion onset) and respective sham-IPost procedures. **b** Summary data illustrating mean ± SD of the densitometry of phospho-AKT-to-AKT ratio. **c** Representative immunoblots showing total p44/42 MAPK (ERK1/2) and phospho-p44/42 MAPK (ERK1/2) (Thr202/Tyr204) protein expression in left ventricular lysates following immediate IPost, early IPost, delayed IPost and respective sham-IPost procedure. **d** Summary data illustrating mean ± SD of the densitometry of phospho-p44/42 MAPK-to-total p44/42 MAPK ratio. **e** Representative immunoblots showing total STAT3 and phospho-STAT3 (Tyr705) protein expression in left ventricular lysates following immediate IPost, early IPost, delayed IPost and respective sham-IPost procedure. **f** Summary data illustrating mean ± SD of the densitometry of phospho-STAT3-to-STAT3 ratio

To determine whether activation of RISK and SAFE pathways is responsible for IPost cardioprotection, we compared the infarct sizes in rats subjected to 30 min of LAD occlusion followed in 10 s or 10 min by IPost in control conditions (injections of vehicle) and after systemic administration of ERK1/2 inhibitor PD-98059, PI3K inhibitor LY-294002 or JAK/STAT pathway inhibitor AG490 (Fig. 2b). In the vehicle-treated group, infarct size was 43 ± 3 %. Both PD-98059 and LY-294002 abolished cardioprotection induced by immediate IPost applied 10 s after ischaemia (infarct size 44 ± 4 and 41 ± 3 %, respectively, both *P* < 0.01 vs IPost10″, Fig. 2b) but had no effect on cardioprotection conferred by early IPost applied 10 min after reperfusion onset (infarct size 24 ± 3 and 23 ± 4 %, respectively, both n.s. vs IPost10′, Fig. 2b). AG490 had no effect on cardioprotection conferred by either immediate or early IPost (infarct size 25 ± 3 and 25 ± 6 %, respectively, both n.s. vs IPost10″ and IPost10′, respectively, Fig. 2b). Western blot analysis revealed significant stimulatory effect of immediate IPost (10 s of reperfusion) on AKT and ERK1/2 (p42/p44 MAPK) phosphorylation (Fig. 3). Early (10 min of reperfusion) or delayed (45 min of reperfusion) IPost had no effect on phosphorylation of AKT, p42/p44 MAPK or STAT3 (Fig. 3).

### The role of mitoK_ATP_ channels in cardioprotection established by IPost

**Fig. 4:**
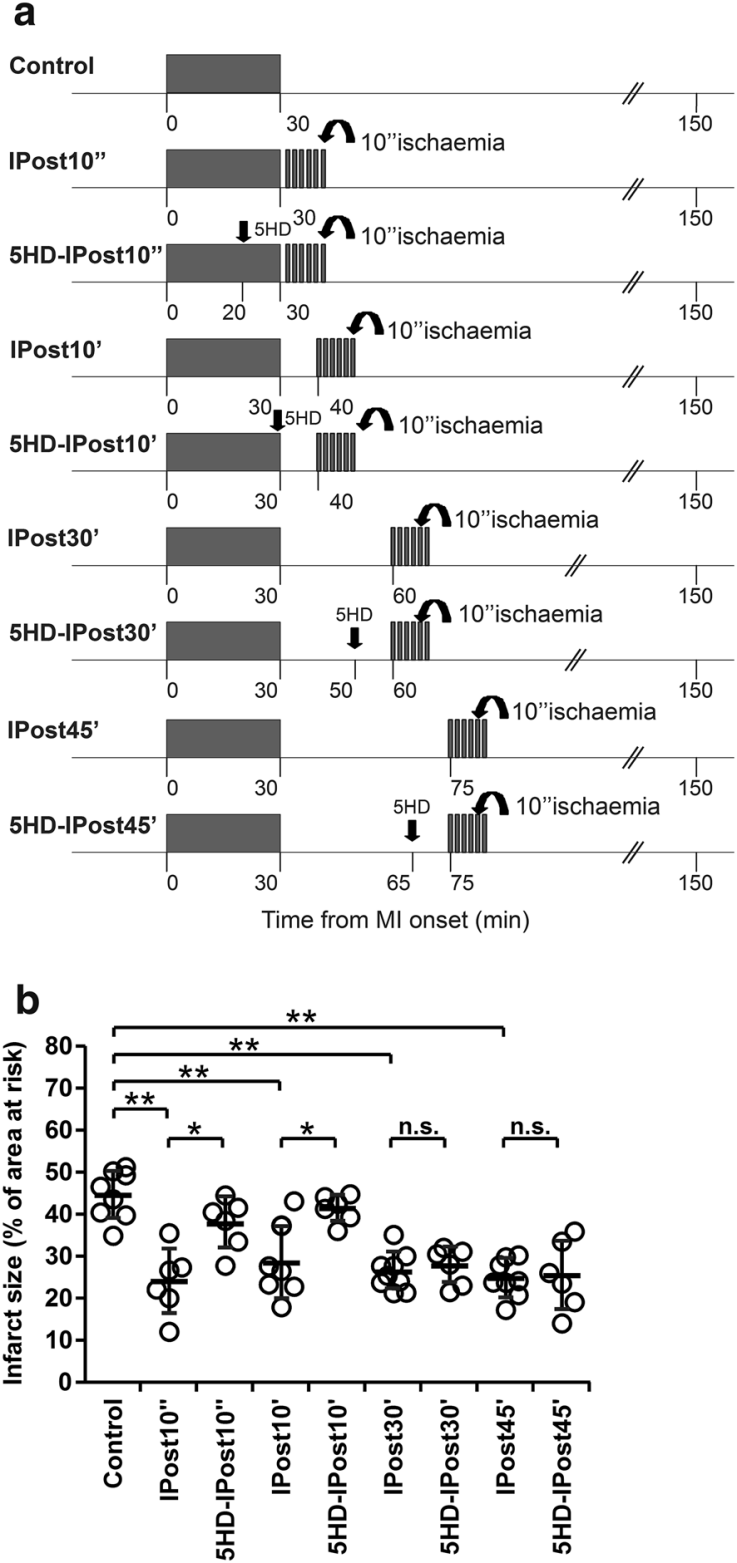
**a** Illustration of the experimental protocols. *Arrow* indicates time of intravenous administration of the mitochondrial K_ATP_ channel blocker 5-hydroxydecanoate (5-HD). **b** 5-HD abolished cardioprotection established by immediate (IPost10″) and early (IPost10′) IPost, but had no effect on cardioprotection conferred by delayed IPost (IPost30′and IPost45′). Infarct sizes are presented as percentages of the areas at risk. Individual data and mean ± SD are shown. **P < 0.01; *P < 0.05; n.s.—not significant

To determine if the activity of mitoK_ATP_ channels contributes to IPost cardioprotection, we compared the infarct sizes in rats subjected to 30 min of LAD occlusion followed in 10 s, 10 min, 30 min or 45 min by IPost (Fig. 4). In the vehicle-treated group, infarct size was 44 ± 5 %. 5-HD abolished cardioprotection induced by IPost applied 10 s or 10 min after ischaemic period (infarct size 37 ± 6, and 41 ± 3 %, respectively, both *P* < 0.05 vs IPost10″ and IPost10′) but had no effect on cardioprotection conferred by delayed IPost, applied 30 or 45 min after reperfusion onset (Fig. 4b).

## Discussion

This study reveals the existence of distinct mechanisms underlying cardioprotection induced by immediate, early and delayed postconditioning. We confirmed in a rat model of myocardial infarction that delayed IPost confers significant cardioprotection even if applied 45 min after reperfusion onset. Efficacy of cardioprotection induced by IPost is critically dependent upon the duration of the prior ischaemic insult. Cardioprotection induced by immediate and early postconditioning involves recruitment of RISK pathway and/or activation of mitoK_ATP_ channels, while delayed postconditioning appears to rely on a different mechanism(s).

### Delayed postconditioning, duration of ischaemia and the importance of early reperfusion for cardioprotective interventions

Since the phenomenon of IPost was first described [51], efficacy of cardioprotection induced by ischaemic postconditioning was demonstrated in various experimental models using mouse, rat and rabbit hearts in vivo and in vitro, as well as dogs and pigs in vivo [40]. However, negative results were also obtained in the experiments conducted in pigs [38] and rats [4, 6, 26]. Although, most of the rat studies which used experimental protocols involving myocardial ischaemia lasting 30–40 min reported significant cardioprotection induced by IPost [43, 44, 48], there are reports that IPost may not be effective in this paradigm [4, 6, 26]. Tang et al. (2006) [44] demonstrated in conscious rats that IPost is capable of conferring cardioprotection only if duration of ischaemia is less than 45 min.

In the present study immediate (applied 10 s into reperfusion period) and early (applied 10 min into reperfusion) IPost effectively reduced myocardial injury only when applied after a period of ischaemia lasting 30 min. IPost had no effect on infarcts which developed following LAD occlusion for either 20 or 45 min. When duration of ischaemia was 30 min, IPost was effective in establishing cardioprotection when applied as early as 10 s and as late as 45 min after reperfusion onset, suggesting that the efficacy of IPost is critically dependent upon the duration of a prior ischaemic period. These data are in agreement with the results of Roubille et al. [35] who demonstrated in a mouse model that IPost confers significant cardioprotection when applied 30 min into the reperfusion. However, these data are discordant with the results of Kin et al. [22] who used a similar MI model in rats and reported complete lack of cardioprotection when IPost was applied 1 min after the onset of reperfusion, suggesting that significant proportion (23 %) of the affected myocardial tissue is irreversibly damaged during the first minute of reflow. The reason(s) for this discrepancy between two studies conducted under similar experimental conditions are unknown. In vivo data which demonstrate the importance of early reperfusion as a window for cardioprotection are surprisingly sparse. Yang et al. [49] reported in a rabbit model of MI that application of IPost 10 min after onset of reperfusion is no longer effective. However, studies in mice [10], rabbits [1], and dogs [8], showed reduction in infarct size by IPost applied 1 min into the reperfusion period.

It has been suggested a decade ago that immediate (very early) period of reperfusion represents the last window of opportunity when cardioprotective treatments could be applied [32]. It is believed that irreversible damage of cardiomyocytes is triggered by opening of the mPTP, which occurs during the first minutes of reperfusion and plays central role in lethal reperfusion injury [1, 5, 10, 13, 19]. Accordingly, preventing mPTP opening either pharmacologically [3, 10] or by application of IPost [1, 29] should reduce lethal myocardial reperfusion injury. However, conflicting data were obtained when mPTP opening was blocked pharmacologically. mPTP inhibitor cyclosporine A failed to reduce infarct size when administered just before the onset of reperfusion in rats [4] and pigs [21]. Opposite data were obtained in humans [31] as well as pig [39] and mouse [3] models which demonstrated reduction in infarct size following cyclosporine A administration.

Recent study suggested that mPTP opening and its role in reperfusion-induced cell death may be dependent upon the duration of the prior ischaemic insult [37]. This study demonstrated that mPTP opening mediates lethal myocardial injury following prolonged periods of ischaemia (50 min), while its role appears to be less significant when the duration of ischaemia is limited to 30 min. These data suggest that death of cardiomyocytes induced by shorter periods of ischaemia followed by reperfusion may be triggered by mechanisms other than mPTP opening. This idea is supported by the results of another recent study which suggested two distinct phases of reperfusion injury [33]. It was reported that following an acute MI, lactate dehydrogenase is released in two separate peaks occurring 2–20 min and 30–120 min of reperfusion, suggesting the existence of two different phases of reperfusion injury. Significant correlation was only observed between the second peak of lactate dehydrogenase release and the resultant infarct size.

Together, recent evidence and our results indicate that early period of reflow may not be the last window of opportunity for cardioprotection and under certain conditions myocardial injury can be reduced by procedures/treatments applied with a significant delay after the reperfusion onset. The data support the idea of a “dynamic wavefront of reperfusion-induced cell death” proposing that lethal myocardial injury develops over time during the reperfusion period [35]. In support of this view, we recently demonstrated in a rat MI model that remote ischaemic postconditioning (femoral artery occlusion) applied 10 min after the onset of reperfusion is also effective in reducing infarct size [2].

### The role of RISK, SAFE pathway(s) and mitoK_ATP_ channels in the mechanisms underlying delayed postconditioning

Recruitment of PI3K-AKT and the p42/p44 MAPK (ERK1/2), known as RISK pathway, follows activation of sarcolemmal receptors by G-protein-coupled ligands or growth hormones [12]. This results in mPTP inhibition mediated via phosphorylation and subsequent inhibition of glycogen synthase kinase 3b (GSK3β) [20]. Activation of RISK pathway is believed to be central in the mechanisms underlying IPost cardioprotection in rats [14, 48]. In this study, pharmacological blockade of RISK pathway abolished cardioprotection induced by immediate IPost but had no effect on cardioprotection conferred by IPost applied 10 min after the onset of reperfusion. These effects are consistent with the Western blot analysis of survival kinase expression which demonstrated that only immediate IPost (but not early or delayed IPost) facilitates AKT and p42/p44 MAPK phosphorylation. JAK/STAT pathway inhibitor AG490 had no effect on cardioprotection induced by either immediate or early IPost and both postconditioning paradigms had no significant effect on STAT3 phosphorylation—the result consistent with the data reported in the literature [43]. Together, these data suggest that activation of RISK pathway may only contribute to cardioprotection induced by immediate IPost, while cardioprotection established by early and delayed IPost rely on different mechanism(s).

One of the possible mechanisms of IPost cardioprotection may involve recruitment of mitoK_ATP_ channels [30]. In the present study, blockade of mitoK_ATP_ channels (systemic application of 5-HD) abolished cardioprotection established by IPost applied 10 s or 10 min, but had no effect on cardioprotection when delayed IPost was applied 30 and 45 min after the onset of reperfusion. Although the exact role of mitoK_ATP_ channels in cardioprotection is not yet clear [9], our pharmacological analysis of the mechanisms underlying immediate, early and delayed IPost suggests the existence of distinct mechanisms underlying these cardioprotective phenomena.

### Clinical relevance

Results of several published animal studies taken together with the data reported here indicate that the efficacy of IPost in inducing cardioprotection is critically dependent on the duration of the preceding ischaemic insult. This may explain the discordant results of human studies which assessed myocardial damage and showed either a significant reduction in infarct size [25, 27, 46, 47], or no effect [7, 45] of IPost. Lack of IPost beneficial effect could be explained by cardiovascular risk factors, pharmacological treatment(s) and various co-morbidities, as previously suggested [17, 50]. Inclusion criteria in some published clinical studies included duration of ischaemia of more than 6 h [7]. Hedstrom et al. [15] performed a comparative analysis of the first time MI evolution in man and the most common experimental animals. The time to reach 50 % infarct size of the area at risk in pigs was found to be 37 min, in rats—41 min, in dogs—181 min and in humans—288 min [15]. Extrapolating rat ischaemia lasting 40–45 min to humans, a corresponding time to reach 50 % infarct size would be ~5 h. Experimental studies (including the results reported here) suggest that IPost is ineffective in rats if applied after such a long ischaemic episode [40]. Therefore, inclusion of patients with long ischaemic time before immediate percutaneous coronary intervention can potentially make it difficult to reveal the beneficial effect of IPost.

Recent study by Roubille et al. [36] demonstrated that in patients with TIMI 2–3 flow grade on admission, corresponding to a delayed application of the postconditioning algorithm, IPost had no effect on infarct size. The authors mentioned that the mean time from the onset of symptoms to intervention, time to hospital admission and door-to-balloon time were similar between the two patient groups and, therefore, cannot explain the absence of IPost beneficial effect. However, the exact time of reperfusion onset in these TIMI 2–3 flow grade patients cannot be accurately determined. Furthermore, the potential interference of coronary microembolization with reperfusion and reperfusion through a residual stenosis, i.e. gentle reperfusion, with protection by IPost cannot be ruled out [16]. Coronary microembolization with reperfusion and gentle reperfusion, both have an effect on infarct size as demonstrated in animal models. Interestingly, previous study conducted in patients with STEMI and reported by the same group demonstrated that thrombus aspiration had no effect on the beneficial effect of IPost, suggesting that a delay of several minutes does not abolish cardioprotection induced by IPost [27].

In accord with current guidelines of STEMI management, most patients undergo coronary thrombectomy first [42] and this may delay application of the first angioplasty balloon inflation of the IPost algorithm beyond the first minutes of reflow. Noman et al. [28] reported that the association between thrombectomy and reduced long-term mortality is only significant in patients when duration of myocardial ischaemia is less than 180 min. This suggests that thrombectomy may be beneficial only in a subgroup of STEMI patients. Results of the present study suggest that following moderate durations of ischaemia, application of IPost with some delay (due to thrombus aspiration procedure) after the coronary artery reopens might have a significant beneficial effect in reducing the extent of myocardial injury and improving prognosis of the patients.
